# Supplementary material for: Electrodeposition of CoxNiVyOz Ternary Nanopetals on Bare and rGO-Coated Nickel Foam for High-Performance Supercapacitor Application
Source: Nanomaterials (Basel). 2022 May 31;12(11):1894. doi: 10.3390/nano12111894 (PMC9182510; doi:10.3390/nano12111894)
Supplement: Supplementary file 1 [file nanomaterials-12-01894-s001.zip › nanomaterials-1736713-SI.pdf]

nanomaterials

Supplementary Materials

Electrodeposition of $Co_xNiV_yO_z$ Ternary Nanopetals on Bare and rGO-Coated Nickel Foam for High-Performance Supercapacitor Application

Seyedeh Mozhgan Seyed-Talebi [1,*], Mohsen Cheraghizade [2], Javad Beheshtian [3], Chun-Hsiao Kuan [1] and Eric Wei-Guang Diau [1,4,*]

1 Department of Applied Chemistry, National Yang-Ming Chiao Tung University, Hsinchu 300093, Taiwan
2 Advanced Surface Engineering and Nano Materials Research Center, Department of Electrical Engineering, Ahvaz Branch, Islamic Azad University, Ahvaz, Iran
3 Department of Chemistry, Shahid Rajaee Teacher Training University, Tehran, Iran; j.beheshtian@gmail.com
4 Center of Emergent Functional Matter Science, National Yang-Ming Chiao Tung University, Hsinchu 00093, Taiwan
* Correspondence: m.seyedtalebi@nycu.edu.tw (S.M.S.-T.); diau@nycu.edu.tw (E.W.-G.D.)

Citation: Seyed-Talebi, S.M.; Cheraghizade, M.; Beheshtian, J.; Kuan, C.H.; Diau, E.W.G. Electrodeposition of $Co_xNiV_yO_z$ Ternary Nanopetals on Bare and rGO-Coated Nickel Foam for High-Performance Supercapacitor Application. *Nanomaterials* **2022**, *12*, 1894. https://doi.org/10.3390/nano12111894

Academic Editor(s): Jung Woo Lee

Received: 06 May 2022
Accepted: 28 May 2022
Published: 31 May 2022

Publisher's Note: MDPI stays neutral with regard to jurisdictional claims in published maps and institutional affiliations.

Copyright: © 2022 by the authors. Submitted for possible open access publication under the terms and conditions of the Creative Commons Attribution (CC BY) license (https://creativecommons.org/licenses/by/4.0/).

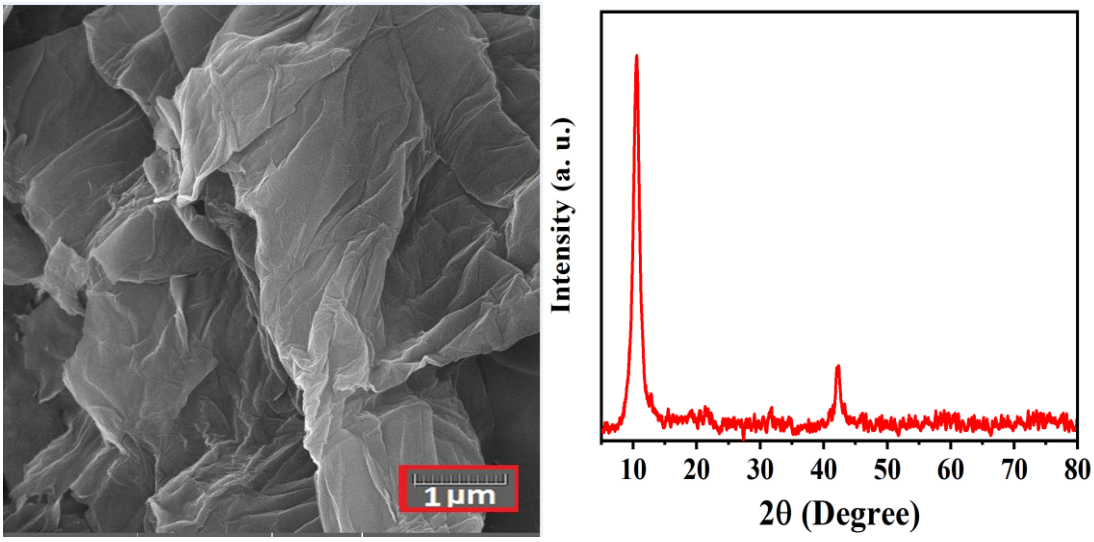

Figure S1. (left) FE-SEM image and (right) XRD pattern of synthesized graphene oxide using modified Hummers method.

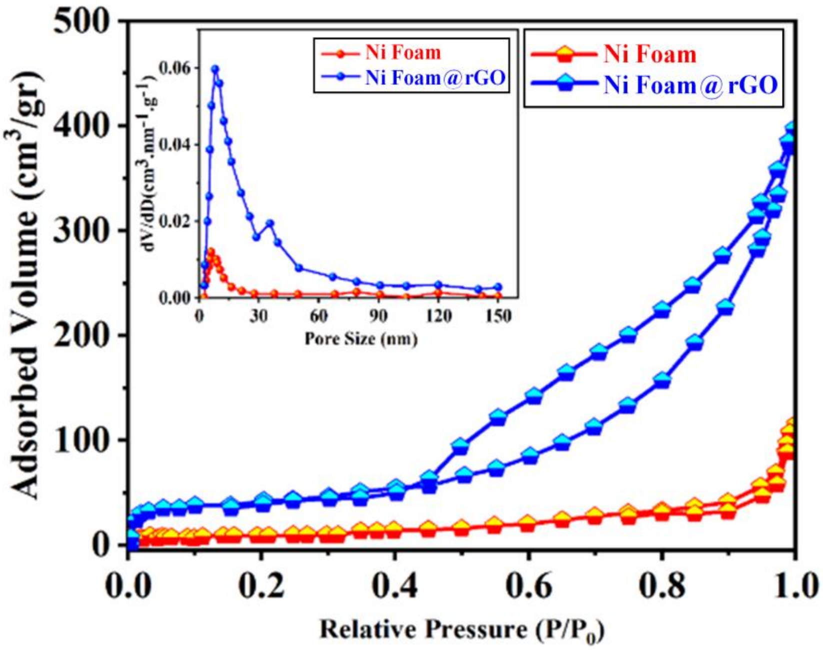

Figure S2. BET surface areas and corresponding distributions of Barrett–Joyner–Halenda (BJH) pore size of coated electrode materials onto Ni Foam and Ni Foam@rGO substrates.

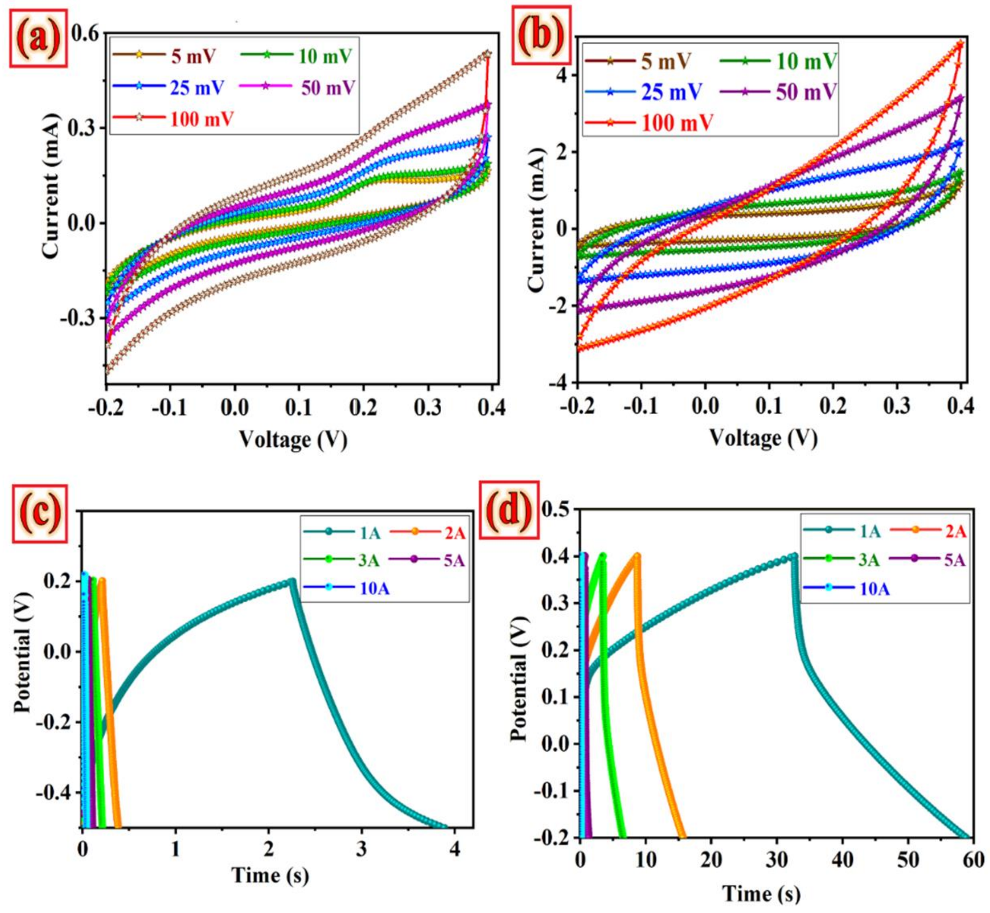

Figure S3. CV curves of typical (**a**) Ni Foam substrate, (**b**) Ni Foam@rGO substrate tested in KOH electrolyte (1.0 M) at scan rates ranging from 5 to 100 mV s^{-1}. Galvanostatic charging-discharging (GCD) curves of a typical (**c**) Ni Foam substrate, (**d**) Ni Foam@rGO substrate at current density ranging from 2 to 150 A g^{-1} in KOH electrolyte (1.0 M).

Table S1. Calculated specific capacitance values of bare, and rGO-coated nickel-foam substrate from GCD tests.

**Current**	**density /A.g^{-1}**	**1**	**2**	**3**	**5**	**10**
**Specific capacitance**	**Ni Foam**	2.63	0.48	0.47	0.35	0.18
	**Ni Foam@rGO**	39.88	23.26	15.4	5.16	1.0

Table S2. Calculated specific capacitance values of $Co_xNiV_yO_z$-based electrodes deposited on bare and rGO-coated nickel-foam substrate from GCD tests.

**Current**	**density/A.g^{-1}**	**2**	**5**	**10**	**15**	**20**	**30**	**50**	**75**	**100**	**150**
**Specific capacitance**	**Ni Foam**	300.31	281.85	263.42	157.92	110.57	99.00	72.14	26.73	5.71	2.14
	**Ni Foam@rGO**	701.08	613.42	584.28	503.57	444.85	348.85	237.85	157.50	25.71	17.14
